# Integrating Social Needs into Health Care: An Implementation Science Perspective

**DOI:** 10.1146/annurev-publhealth-071823-111332

**Published:** 2024-10-30

**Authors:** Maura Kepper, Callie Walsh-Bailey, Constance Owens-Jasey, Rose Gunn, Rachel Gold

**Affiliations:** 1 Prevention Research Center in St. Louis, Brown School, Washington University in St. Louis, St. Louis, Missouri, USA; 2 Department of Medical Social Sciences, Feinberg School of Medicine, Northwestern University, Chicago, Illinois, USA; 3 OCHIN, Inc., Portland, Oregon, USA; 4 Kaiser Permanente Center for Health Research, Portland, Oregon, USA

**Keywords:** social needs, health care, implementation science, sustainability, health equity

## Abstract

Unmet social needs (e.g., housing instability, food insecurity, transportation barriers) impact a patient’s ability to participate in health-seeking behaviors (e.g., physical activity, routine preventive care) and to achieve optimal health. A rapidly growing number of health care systems are incorporating social needs screening and assistance into clinical workflows, yet many implementation and sustainability challenges exist and require collaboration with social service organizations. This review highlights implementation approaches used within this rapidly changing US landscape and uses implementation science frameworks to systematically identify multilevel barriers to and facilitators of implementing and sustaining social needs care. Policies and economic investments are necessary as they determine critical barriers and facilitators within the clinical and social service contexts. Implementation may be further strengthened by cross-sector engagement, evidence-based implementation strategies, and capacity building within clinical and social service organizations. Successful, sustained implementation of social needs care may improve the quality of health care, population health, and health equity.

## INTRODUCTION

Social determinants of health (SDOH)—“the conditions in the environments in which people live, learn, work, play, worship, and age” (1, 62)—have a profound influence on health outcomes and health equity (see the definition quoted from Reference 43a). The SDOH categories from the US Healthy People 2030 framework are economic stability, education access and quality, health care access and quality, neighborhood and built environment, and social and community context (https://health.gov/healthypeople/priority-areas/social-determinants-health). Adverse SDOH are upstream factors that result in individual-level social risks (e.g., housing instability, food insecurity, transportation barriers), which are called social needs when the patient prioritizes their mitigation (3). Ample evidence shows that social risks impact one’s ability to participate in health-seeking behaviors (e.g., physical activity, routine preventive care) and to achieve optimal health, which leads to health disparities and health inequities (3).

The convergence of this evidence with emergent value-based care incentives is driving the health care sector to better integrate with social service organizations (SSOs) (55, 83). A 2019 National Academies of Science, Engineering, and Medicine (NASEM) report identified five activities for health care sector integration of social needs care—awareness, adjustment, assistance, alignment, and advocacy—which has generated recommendations for how to conduct such interventions, to implement policy changes, and to develop new technologies (59). Emergent evidence demonstrates the potential for awareness and assistance interventions to improve access to community resources, reduce health care spending and utilization, and improve health (96), and a relatively large body of evidence has documented the successes and failures of US health care systems in implementing awareness and assistance interventions. This review focuses on awareness and assistance activities.

Awareness activities typically involve using screening tools (42) to identity patients’ social risks and unmet needs, as well as determining who conducts screening, which patients receive screening, and how frequently they receive screening; these decisions require careful planning and integration into routine practice to ensure successful adoption and sustainment. Awareness generated from these processes may then direct the provision of assistance, as well as other interventions (e.g., adjustment of care) not discussed in this review. Assistance interventions may include internal referrals to connect the patient with clinic staff members [e.g., community health workers (CHWs), social workers, navigators] who provide direct assistance (e.g., a food box) or support them in accessing a resource in the community (e.g., food pantry) or in obtaining external referrals to local SSOs (59). Clinics may use one or a combination of referral methods (67). Policies and reimbursement models (e.g., Medicaid, Medicare Advantage) are expanding coverage to include care coordination, including managing social needs; electronic health record (EHR) vendors are incorporating social risk assessments as standard modules; and new technologies are enabling electronic referrals to SSOs (34).

While awareness and assistance interventions are becoming more common in clinical settings (47), these activities still occur in a minority of settings, and they have been adopted by certain practices more than others (e.g., those with payment or primary care improvement models). Even when such activities are adopted, their equitable and sustained implementation (i.e., reaching and meeting the needs of all patients over time) is a substantial challenge in many care settings (35, 47). Implementation science can be leveraged to enhance the adoption, implementation, and equity of effective practices by (*a*) systematically identifying key factors influencing the success of social needs awareness and assistance interventions; (*b*) supporting the design and adaptation of interventions for optimal alignment with recipient needs and preferences; (*c*) guiding the selection and tailoring of implementation strategies to enhance such programs’ integration into clinical care; and (*d*) using well-tested approaches to plan for the sustainability of social needs interventions (20, 21, 69, 80).

To our knowledge, no prior articles have utilized implementation science to inform a structured, multilevel approach to integrating social needs care into routine health care. This review highlights some implementation approaches used within a rapidly changing US landscape and uses implementation science frameworks to systematically reflect on the diverse factors that impact successful and equitable implementation and sustainment of social needs care within health care systems. While this article focuses on the United States, its findings are likely applicable to other countries.

## ORGANIZING FRAMEWORK

**Figure 1:**
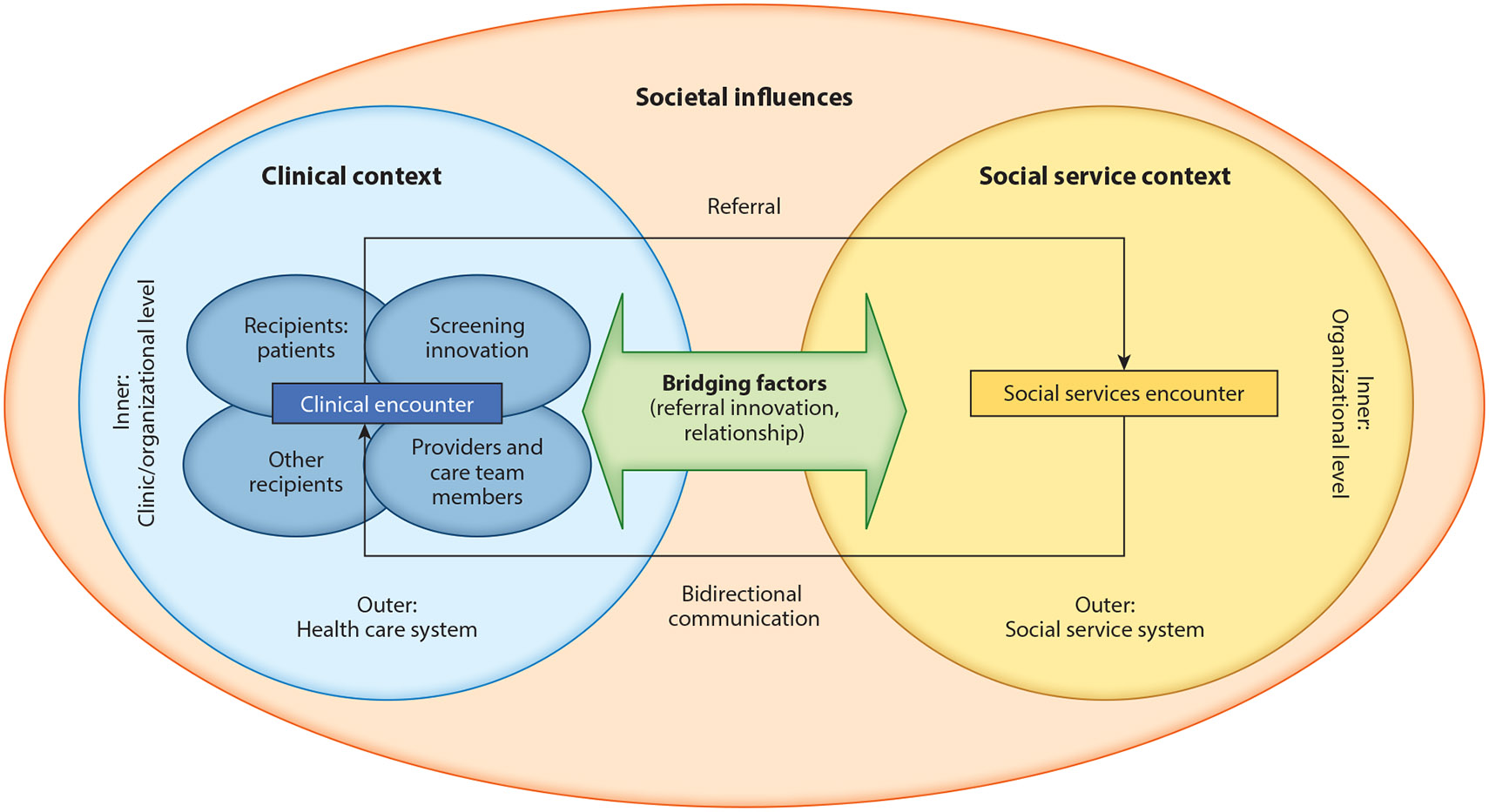
The social care implementation framework. Figure adapted from the Health Equity Implementation Framework (HEIF) (94) and the Exploration, Preparation, Implementation, Sustainment (EPIS) framework (1).

This review combines the Health Equity Implementation Framework (HEIF) (94) and the Exploration, Preparation, Implementation, Sustainment (EPIS) framework (1) to generate a social care implementation framework (Figure 1). This framework is used to guide the exploration of the multilevel determinants relevant to the equitable and sustainable implementation of social needs awareness and assistance interventions (see the definition for Determinants from Reference 60). The HEIF considers factors related to the uptake of an innovation (here, social needs awareness and assistance interventions) in health care, accounting for the interaction between the patient and the provider during the clinical encounter and factors at multiple levels: the clinic organizational level (inner context), health care system level (outer context), and societal level. Four categories (characteristics of the innovation, patient factors, provider factors, and other recipients) intersect to create an individualistic innovation opportunity for the patient (i.e., the clinical encounter) that is affected by the inner (clinic/organizational level) and outer (health care system) contexts (94). Given this review’s focus on social needs assistance, including referrals to SSOs, EPIS was added to include bridging factors that identify critical aspects of the health care and social service relationship. This combined conceptual framework includes the inner and outer settings of SSOs as these contexts impact an organization’s capacity to meet patients’ needs and improve health outcomes. Both the health care and social service inner and outer contexts are impacted by societal influences, which the HEIF identifies as economies, physical structures, and sociopolitical forces. Because this framework focuses on equity, the consideration of inner, outer, and societal contexts is intended to account for historical and ongoing racial, social, economic, and political marginalization (31).

## KEY DETERMINANTS AND CONSIDERATIONS FOR IMPLEMENTATION

The sections below discuss determinants (summarized in Table 1) and key considerations for implementing and sustaining awareness and assistance interventions. They are organized by using the social care implementation framework (Figure 1) to systematically consider these factors across levels and clinical and social service contexts. The factors influencing the success of social needs awareness and assistance interventions may be barriers or facilitators. While several known determinants are presented, the list is not comprehensive; substantial gaps remain in our understanding of the complex factors that impact awareness and assistance intervention implementation in clinical settings (89).

### Societal Influences

Societal influence includes the sociopolitical forces and physical structures that have a downstream influence on the innovation, recipient, and inner and outer contexts. As depicted in Figure 1 all factors related to the implementation of awareness and assistance interventions are influenced by the societal level.

#### Sociopolitical forces.

Several studies demonstrate how public policies may influence the implementation of awareness and assistance interventions (48, 63, 86, 87). New social care quality measures implemented by the National Committee for Quality Assurance, the Centers for Medicare and Medicaid Services, and others require providers to document and track the proportion of patients screened and, in some cases, the resources or interventions provided. Medicaid 1115 waivers allowing clinics to be reimbursed for the provision of social supports, such as housing assistance and food, have incentivized US health care systems to screen, document, and track referrals for social needs. State and federal policies regarding reimbursement from public payors, particularly Medicare and Medicaid, determine whether assistance efforts are reimbursable, for which staff, and at what amount (48, 63, 86). Policies around data security, privacy, and confidentiality can also restrict cross-sector information sharing to coordinate referrals and service provision (57, 87). Policies and economic investments create barriers or facilitators described within the clinical and SSO inner settings (e.g., staffing, infrastructure), as well as the physical structures (e.g., access to transportation and SSOs) described below. One overarching challenge to the equitable implementation of social risk efforts is the variation in what each state’s Medicaid system covers.

Beyond policy, societal-level stigmas, discrimination, or pressures can impact patients’ willingness to disclose social needs and seek assistance (23, 85). Cultural norms may also impact a patient’s decision to declare social needs and receive assistance (66).

#### Physical structures.

Community-level SDOH driving social risks differ across neighborhood environments. Thus, even if a patients accepts a referral, those in neighborhoods with limited infrastructure (e.g., public transportation) may face barriers to accessing and utilizing resources (67). Rural communities, for example, have limited public transportation, availability of primary care and specialty services, and SSOs (77). These resource disparities in physical structures, as well as an overall national shortage of SSOs, are driven by chronic underfunding, i.e., policy decisions, and impact the implementation of awareness and assistance interventions.

### Clinical Context

The clinical context includes the people (patients and care team members), innovation, and inner and outer clinic and health care system settings that influence the clinical encounter in which an awareness and assistance intervention is delivered.

#### Recipients (patients).

Patients generally report acceptability of social needs screening and comfort in discussing social needs with their care team (4, 7, 14, 19, 24, 27, 78, 89, 91, 92). However, patients who experience greater needs are more likely to express discomfort with screening compared to those with no or fewer social needs (7, 24, 91), and a high proportion of patients who report social needs decline assistance to address those needs (6, 7, 14, 85, 91). Previous health care experiences, including those related to screening and assistance, influence these perspectives (23, 24). Positive prior experiences with screening, including perceived improved care quality and receipt of beneficial resources, facilitate patient receptivity (27, 89). Uncertainty of how screening information is used and documented in the medical record, discomfort disclosing sensitive information, fear of being judged, or past experiences of health care discrimination reduce patients’ acceptance of screening and assistance (23, 24, 41, 57, 82, 89, 92). Historical and current medical mistrust and systemic racism have disproportionate implications for the willingness of persons of color to report their social needs (8). When screening pediatric populations, parents may fear being reported to child protective services if they disclose social risks such as food insecurity (58). As noted above, perceived stigma can influence patients’ willingness to receive assistance. The quality of the relationship with their provider is another prominent determinant to receptiveness of or hesitancy toward reporting social needs and receiving assistance (27, 84, 89).

#### Recipients (providers and care team members).

Provider attitudes and beliefs toward screening and assistance are frequently reported. Provider awareness of the impact of social needs on patients and perceptions that screening is appropriate and beneficial and can improve care are prominent facilitators (27, 41, 43, 51, 54, 62, 63, 72, 75, 89, 90, 92). Common challenges to screening include low comfort or self-efficacy with screening and discussing social needs with patients (7, 10, 41, 54, 72, 88, 90). Providers often express concerns about negative patient reactions to screening and are worried about causing discomfort or distress by asking about social needs (33, 54, 72, 88, 90). Lack of knowledge and awareness about SSO resources and perceptions of or knowledge that such resources are unavailable, inadequate, or poorly aligned with patient needs are also common barriers (7, 41, 43, 51, 72, 87). Providers do not want to ask about needs that they feel they cannot address. Positive experiences with screening and influential peers who hold favorable views of screening are facilitators that can help alleviate provider concerns (19, 63, 72, 89, 92). The extent to which providers view that social care is aligned with clinical goals and fits within providers’ own role is also an influential determinant (19, 43, 54, 63, 72, 92).

#### Characteristics of the screening innovation.

Identifying unmet social needs in clinical settings commonly involves using screening tools; numerous low-cost, low-literacy tools are available (42). The simplicity of the screening tool and associated administration processes are determinants of successful implementation. Clear, simple items written at an accessible literacy level, in the patient’s preferred language, and use of images and visual aids can facilitate screening (10, 15, 27, 41, 43, 82). Yet psychometric data are limited, and social needs screening tools are often available only in English (42). The PRAPARE tool is an exception, with availability in 32 languages (https://prapare.org/). Screeners with questions that ask patients which social risks they want or need help with (i.e., unmet social needs) facilitate appropriate assistance interventions.

Characteristics of the screening workflow, such as whether screening is conducted in a private location and whether patients can complete the screening without interrupting the flow of the clinic visit, can also facilitate screening (85, 88, 89). The quality of evidence for screening is also an influential determinant because convincing evidence for the screening approach can support screening and a lack of evidence or doubts about evidence quality can be barriers (90, 92) (see the sidebar titled Review of Screening Tools).

#### Inner setting.

Well-planned integration of screening tools and practices into existing systems and workflows, including the EHR, can improve the ease of screening and subsequent assistance interventions; misalignment with current practices presents a barrier (43, 72, 88). Staff availability and capacity influence the success of social needs awareness and assistance interventions. The presence of dedicated staff to administer and monitor patient screening is a key facilitator (22, 54, 57, 62-64). Competing demands and limited time during the clinic visit make it challenging for physicians or other providers to both conduct screening and address identified needs (13, 27, 41, 51, 54, 72, 76, 86, 88, 89). Organizations that do not have dedicated team members (e.g., social workers, CHWs) with expertise in properly documenting social needs and providing assistance generally have trouble implementing and sustaining practices (51, 54, 57, 65). Funding for adequate staffing, often driven by policy, is a key organizational determinant (57, 65). CHWs and social workers who have cultural and linguistic congruence and can build rapport with patients are ideal care team members to provide social needs care, so increasing pathways to funding and sustaining this staff is critical (46, 74, 79). These barriers may be particularly prominent in clinics in rural and underresourced urban communities that face staffing shortages (11).

Clinic leadership must buy in to the importance of social care integration and foster an organizational climate that prioritizes social care (13, 65, 88). Leadership support depends on factors including perceptions of such efforts’ importance, available resources, rising costs of delivering care, regulatory and reporting requirements, and the need to adapt operations and provide ongoing training (59).

### Bridging Factors and Referrals

Bridging factors acknowledge the interrelated nature of the clinic and social service contexts in implementing assistance interventions. Bridging factors include characteristics of the referral innovation and relationship between health care and SSOs.

#### Characteristics of the referral innovation.

Social service resource locator (SSRL) systems such as UniteUs and FindHelp are promising emergent tools for supporting assistance referrals (28). These technologies are intended to identify local, state, and national resources and manage and track referrals. However, SSRLs have limited capacity for maintaining resources or access to universal databases to maintain resource information, placing the burden on local SSOs to maintain resource offerings and increasing the administrative burden of health care teams to verify services. Furthermore, SSRLs often charge users for bidirectional referral and communication capabilities between organizations, which hampers closed-loop communication and tracking across health care organizations and SSOs to ensure that patient needs are met. SSRLs that are effectively embedded in existing systems and workflows within health care organizations and SSOs can facilitate more successful assistance interventions; however, the misalignment of their use with current practices presents a barrier to referral-making (43, 72, 88) (see the sidebar titled Guide for Selecting a Referral Platform).

#### Relationships.

Effective social risk referral-making requires collaborations between health care teams and SSOs to ensure that patients are connected to appropriate services and successfully receive those services. An emergent approach to establishing these connections involves bidirectional referrals, also known as bidirectional communication and referral pathways (BCRPs) (25). While BCRPs are ideal, implementing them involves bridging health and social service silos and thus cross-sector systematic process changes (25). High-quality relationships, effective communication, and shared goals across partners facilitate assistance interventions (27, 57, 65). Health system fragmentation, a lack of service agreements, and the inability to efficiently communicate and coordinate hinder referral programs (27, 57, 65).

### Social Service Context: Inner Setting

SSOs provide specialized services to support social needs and often have strong ties to and trusting relationships with the communities they serve. Yet they are chronically underfunded, creating workforce shortages and overall limited resource availability (38). Other barriers in this setting, often shaped by public policies, include SSOs’ service quality, relevance, long waitlists, eligibility requirements, and capacity (27, 43, 54, 75, 85, 96). As a result, health care providers may be unsure that making a referral will result in patients’ needs being met and thus may choose not to screen patients or offer referrals.

## CASE EXAMPLES: IMPLEMENTING SOCIAL NEEDS AWARENESS AND ASSISTANCE INTERVENTIONS

This section provides examples of social needs awareness and assistance interventions. Each example provides findings that highlight barriers and potential strategies for implementing and sustaining social needs care in different health care settings.

### Case Example 1: Integrating Social Determinants of Health Screening and Referral During Routine Emergency Department Care: Evaluation of Reach and Implementation

The first case example took place in an academic level 1 trauma center emergency department (ED) in Utah (92). As a facility in the US health care system where patients cannot be turned away for an inability to pay, EDs care for a disproportionate number of low-income and uninsured patients.

#### Screening and assistance approach.

The ED registration staff, who routinely interact with patients to obtain contact and insurance information after ED admission, were trained to administer the Screener for Intensifying Community Referrals for Health (SINCERE), a 10-item, low-literacy screening tool available in English and Spanish. Staff administered this screener via a REDCap electronic form from January 2019 to February 2020 as part of their registration workflow using touchscreens. The screenings generated automatic referrals to resource specialists at the United Way 2–1–1 program (211), who followed up with patients using their preferred contact information after the clinical encounter. The Utah 211 is staffed by trained resource specialists with access to information on more than 10,000 services in Utah and surrounding states. Resource specialists are subject to routine quality oversight and use Health Insurance Portability and Accountability Act (HIPAA)-compliant software to track service use, consumer demographics, reported needs, and consumer follow-up. Information from the 211 database was routinely imported back into the University of Utah REDCap using a unique patient identifier.

#### Evaluation approach.

A convergent mixed-methods design using the RE-AIM (Reach, Effectiveness, Adoption, Implementation, and Maintenance) framework (32) was applied. Reach was evaluated as the number of patients who were approached, completed screening, and received service referrals. Adoption was assessed qualitatively as the receptiveness of patients and providers to engage in screening and outreach service referrals. Data were gathered via the REDCap database, observations, staff interviews, and patient focus groups.

#### Findings.

Of the 61% (*n* = 2,821) of approached patients (*n* = 4,608) who completed screening, 47% (*n* = 1,324) reported one or more social needs. About 7% (98/1,324) of patients with a reported need were reached by 211 staff and received a referral to an SSO, most commonly for utilities services, rent payment assistance, and food pantries. ED registration staff tailored the screening administration based on their own professional intuition, felt unsure if the screener was the appropriate tool, and questioned the usefulness of screening as part of their staff role. Those who had intrinsic motivation (e.g., felt their role served a bigger purpose in patients’ lives) were more likely to screen. Overall, patients felt positive about the screening yet noted the potential for embarrassment and the need for sincerity from the care team. Patients suggested that staff use strategies to show that they care, such as making eye contact, and preferred to be asked by a staff member they knew. There was some indication of low assistance reach, which illustrates the need to understand factors beyond screening and referrals, such as decisions to accept outreach and ultimately to act on referrals.

### Case Example 2: Implementation of EHR-Integrated Community Resource Referral Platform to Support Social Needs Care in a Community Health Center System

The second case example took place in an urban community health center (CHC) that provides comprehensive primary care services, including medical, behavioral health, and dental care throughout the lifespan (40). Its four clinics serve more than 20,000 patients. The CHC participated in a pilot project focused on implementation of an SSRL integrated with their EHR (40). The CHC had already been systematically screening for social risks and using a nonintegrated SSRL when this pilot project began.

#### Screening and assistance approach.

Patients were screened for social risks during rooming. If patients reported having a social need, a navigator connected with them at their clinic visit via a warm handoff or followed up by phone afterward. Navigators identified resources and made referrals to community and nationwide resources within the EHR using the Findhelp (29) SSRL.

#### Implementation support.

Implementation support and evaluation were led by the host of the CHC’s EHR, a nonprofit health IT organization (i.e., OCHIN). OCHIN provided technical support for SSRL–EHR integration and implementation, which started with a virtual training on using the integrated SSRL. An experienced practice coach conducted monthly facilitation calls with clinical and project champions to assess site readiness and identify potential barriers and facilitators, to build a coalition of implementation partners, and to develop a formal implementation blueprint. The project champion led staff training sessions in motivational interviewing techniques to bolster the communication skills of key staff who were involved in screening and referrals (e.g., medical assistants, navigators). OCHIN developed and implemented a tool for monitoring the quality of screening and referral data, which was used to provide feedback in monthly coaching calls.

#### Evaluation approach.

A formative mixed-methods evaluation was conducted between February 2021 and June 2022 to understand implementation and use of the screening program and EHR-integrated SSRL. This evaluation included a combination of quantitative data and qualitative data from CHC staff and leadership interviews and surveys, observation of implementation meetings, and utilization data extracted from the EHR and SSRL platforms.

#### Findings.

By the end of the 10-month pilot period, the CHC had used the integrated SSRL to provide SSO information to 287 patients, primarily for food and housing. While resource information was searched for via the integrated SSRL, referrals were rarely placed directly through the platform, largely due to few SSOs accepting SSRL-based referrals. Though referrals were documented in the patient chart, referral status was often not tracked due to time constraints. Despite the limited referral functionality and annual integration maintenance costs, the CHC reflected positively on its participation in the pilot project because of the robust SSO database, added efficiencies inherent in the SSRL embedded in the EHR, and implementation support activities that improved the CHC’s screening and referral workflows.

### Case Example 3: Integration of Community Resource Specialist on Primary Care Team for Social Needs Navigation

This case example included 32 primary care clinics within an integrated health care system across Washington state, serving more than 600,000 patients in urban, suburban, and rural communities (2, 26, 61).

#### Screening and assistance approach.

A community resource specialist (CRS) role was codeveloped with patients, primary care team members, health system operations staff, and researchers in a pilot project in four clinics. Following this pilot, the health system invested in full-time CRSs across all primary care clinics. The CRS serves three primary functions: (*a*) Screen patients for social needs, (*b*) provide tailored assistance to resolve social needs, and (*c*) develop and maintain relationships with SSOs in the community. The Your Current Life Situation (YCLS) screening tool (44), developed by Kaiser Permanente, was used to screen for social needs through the EHR. Patients with known or suspected social needs received an EHR referral or warm handoff from a clinician to the CRS, or patients could request a CRS appointment. CRSs tailored assistance approaches to meet the needs of the patient; approaches included providing resource referrals (information or direct connection to resources), goal setting and action planning, and continued follow-up to close the loop on patient needs.

#### Implementation support.

As part of the organization’s learning health system initiative, researchers and clinical operations partners collaborated to develop processes to integrate the CRSs and evaluate the implementation. Key implementation strategies included practice facilitators to support workflow design, EHR technician support for integrating the YCLS screening tool into the EHR, a robust CRS training program, supervision, and regular CRS team meetings to facilitate peer support.

#### Evaluation approach.

A mixed-methods summative evaluation was conducted from 2019 to 2021 to assess the implementation of the CRS role and its impact on patient satisfaction. The evaluation used propensity score matching to compare patients with multiple, one, or no CRS encounters. A combination of quantitative and qualitative patient and care team data was collected from the EHR, surveys, interviews, and site visits.

#### Findings.

Of the 1,159 patients enrolled in the study, 384 had a single CRS encounter, and 354 had 2 or more CRS visits. Patients with multiple visits reported greater satisfaction with the CRS and overall trust in their health care team than did patients with a single CRS visit. Implementation challenges included a lack of clinician knowledge about the CRS role and about how to refer patients to the CRS, as well as inconsistent workflows for screening referrals, screening administration, documentation, and follow-up to determine if patients’ social needs were adequately addressed. Facilitators supporting CRS integration included financial investment from the health system, leadership support, a dedicated manager to supervise the CRS team, and a supportive team structure.

## POTENTIAL APPROACHES TO ENHANCE IMPLEMENTATION

Societal influences, mainly policies and associated funding, are key determinants of awareness and assistance efforts in the clinical and social service contexts and, therefore, are critical to improving social needs care. Policies must provide reimbursement for social needs care in clinical care settings and funding for clinical and social service organizations to provide the staffing, infrastructure, and resources required to effectively integrate awareness and assistance interventions. Enactment of a policy does not ensure that it achieves its intended benefit; the implementation science field must also support and measure the successful adoption, implementation, and sustainment of policy actions to ensure that benefits are realized (5, 18).

While societal influences drive many of the inner setting factors that determine the success of social needs care, use of evidence-based implementation strategies—the diverse actions, techniques, and tools used to support practice change (49, 68, 70, 93)—to optimize successful implementation within the clinical and social service contexts also remains necessary. Using the social care implementation framework presented here to consider these barriers to and facilitators of social needs awareness and assistance may help researchers to more systematically understand critical barriers (e.g., provider knowledge, attitudes, and beliefs; workflow integration; organizational priority and leadership support) that must be addressed to improve social needs care for all patients over time. Future work should both build from our social care implementation framework and leverage advances in integrating equity into implementation science methods and measures to confirm equitable implementation and outcomes of social needs care (9, 12, 81, 94, 95).

Evidence-based implementation strategies were proposed in the NASEM report targeting the clinic-level context (patients, clinic teams, workflows) and bridging factors (referred to in the report as community partnerships) (59). At the time of this report, almost none of the strategies had been assessed specifically for social needs care (59). Subsequent research, albeit still limited, has tested some of these strategies. A large-scale study in 31 CHC clinics tested the impact of implementation strategies on social risk screening and referral rates (36). Strategies included receiving technical assistance and practice coaching, which supported clinics through a five-step implementation process that included securing leadership buy-in and developing workflows. While these strategies increased screening rates during the intervention period, these effects were not seen for referral rates and were not sustained post-intervention (36). Our case examples highlight the potential benefit of clinic champions, training, and practice facilitation strategies (40, 61). Selecting strategies based on research-informed determinants (those outlined in this review) that are context specific is likely necessary to meet the needs of specific clinic settings. Using participatory approaches that engage end users such as the clinic staff who will implement the strategy, patients, and organizational leaders to help implementers understand contextspecific determinants and select and operationalize strategies may also improve the impact of these approaches. This process may involve working with diverse partners to operationalize each strategy by defining it and naming components such as the actor, action targets (recipients and intended changes among them), and implementation outcomes (e.g., increased feasibility and fidelity) critical to a strategy’s success (71). Understanding the mechanisms by which strategies enact change is another critical step because it helps to identify the determinants being addressed and the conditions in which strategies work well (56). Understanding mechanisms for change may ultimately improve the ability to tailor these strategies and to scale up these approaches to support social needs care across different clinic settings.

Effectively integrating social needs care into the workflows of clinics and SSOs is an important part of successful implementation. Participatory design approaches (such as codesign, community-based participatory research, community-engaged research) and process mapping methodologies (17) may enable clinic systems, SSOs, patients, and implementation scientists to work collaboratively to codevelop screening, referral, and follow-up processes that are feasible within and across health care and SSO workflows (30). Using participatory approaches may generate buy-in and flexibility around workflows and align practices with clinic needs, interests, and resources (39). Involving SSOs in this process is critical because barriers to addressing social needs arise from the lack of a quality relationship and shared communication and goals (bridging factors), and workflows should be adjusted based on SSO inner-setting factors (e.g., availability of resources). Patient engagement is equally important and may help to improve the equity of social needs care by having patients inform how screening is conducted, such as who conducts the screening, which could increase their comfort level for sharing sensitive information and reduce the fear of judgment and discrimination (recipient-level determinants). While adjusting workflows may improve patients’ comfort, societal influences such as culture, stigma, and discrimination, as well as historical events that have caused medical mistrust among marginalized populations, are at the root of recipient attitudes and beliefs that can be barriers to implementation.

Modifications to workflows and practice may be tested using quality improvement (QI) initiatives and small tests of change such as plan, do, study, act cycles (73). These tools are commonly used among one team or one site over the course of a short, specified timeframe (e.g., one month). In this way, potentially costly investments are not adopted and scaled up until and unless the pilot strategy proves effective. QI programs are already integrated and institutionalized with buy-in from team members at all levels of the organization due to regulatory requirements (i.e., from Health Resources and Services Administration (37) and Medicaid health care reform initiatives) for comprehensive work plans and key performance indicator reporting. While these methodologies are not specific to social needs care, the use of participatory methods and implementation research initiatives that build on the QI model can enhance efforts to effectively implement social needs referral and assistance programs at scale (73) (see the sidebar titled Guides to Implementing Screening and Referral Practices).

## SUMMARY AND CONCLUSION

The national drive toward health care systems addressing social needs has hastened related implementation efforts in some clinics and systems. This review highlights implementation findings from this rapidly changing landscape and describes the multilevel determinants identified as impacting the implementation of social needs care within health care systems. The use of implementation science and a new adapted health equity implementation science framework to systematically catalog barriers and facilitators may catalyze future efforts to test evidence-based strategies and approaches for improving the implementation, effectiveness, and sustainability of social needs awareness and assistance interventions.

Implementation science could help develop guidance and evidence regarding the strategies (actions, techniques, and tools) that support the adoption, implementation, and sustainment of social needs care in routine practice (49, 68, 70, 93). Investment in technologies and data systems is essential and must be paired with adequate resources and implementation strategies that support their use. The success of social needs awareness and assistance interventions depends on their acceptance from health care organizations and the health care team. Health care providers need systems that they can trust will address their patients’ needs. The use of participatory methods to develop implementation processes and workflows that meet patients’ needs, and are feasible within clinics and SSOs, may help improve equitable and sustained implementation.

We are at a critical point in time in integrating social needs care into our health care systems. Though many related efforts are underway, many challenges exist, and stronger evidence is needed to support successful implementation and demonstrate impact on population health and health equity.

## Figures and Tables

**Table 1:** Summary of determinants of social needs screening and referral across multiple levels of the social care implementation framework

	Determinants^a^	
	Awareness (screening)	Assistance (referral)
Societal influences		
Sociopolitical forces	Culture, stigma, and discrimination for having social needs (B) Policies, laws, and regulations (B, F) Social care quality measures Public policies regarding staff credentialing and reimbursement	Culture, stigma, and discrimination for receiving help for social needs (B) Policies, laws, and regulations (B, F) Reimbursement waivers (Medicaid 1115) Policies on data sharing, privacy, and confidentiality Chronic underfunding of social safety net (B)
Physical structures	Health care access (B, F)	Limited public transportation (B) Density, reach, and diversity of SSOs available (B, F)
Clinical context		
Recipients (patients)	Knowledge, attitudes, and beliefs perception of screening as acceptable, appropriate (F) uncertainty over how information is used, documented (B) discomfort with sharing sensitive information (B) fear of being judged/discrimination (B) parents’ fear of being reported (B) Previous experiences (positive and negative) with health care, screening (B, F) Quality of relationship with provider (B, F)	Lack of desire to receive referrals (B) Previous experiences (positive and negative) with health care, assistance (B, F) Quality of relationship with provider (B, F)
Recipients (providers and care team members)	Knowledge, attitudes, and beliefs understanding of SDOH and impact of social needs on health (F) belief that screening is important and useful and that it helps improve care (F) beliefs about alignment of screening with professional role (B, F) lack of knowledge of available resources to address social needs/perception of resources as inadequate (B) concern about negative patient perceptions, reactions (B) Skill and experience (B, F) Discomfort, low self-efficacy for screening/discussing social needs (B)	Knowledge, attitudes, and beliefs Uncertainty that patients’ needs will be met (B) beliefs about alignment of assistance with professional role (B, F) Knowledge of resources and personal relationships with SSOs (F)
Characteristics of the screening innovation	Relative complexity (B) or simplicity (F) of screening tool and process Accessible literacy level (F) Language (B, F) Appropriateness of screening tool and modality for patients (B, F) Quality of evidence for screening (B, F)	Inclusion of questions on which social risks the patient desires/needs support with (F)
Inner setting	Extent of alignment between screening practices and routine clinical workflows (B, F) Limited time and competing demands during clinic visit (B) Dedicated staff to support screening (B, F) Funding, investment in staff, infrastructure (B, F) Organizational priority, leadership support (B, F)	Extent of alignment between assistance practices and routine clinical workflows (B, F) Limited time and competing demands during clinic visit (B) No or part-time team members dedicated to assistance (B) Care team members (e.g., social workers, CHWs) with expertise in assistance (F) Funding, investment in staff, infrastructure (B, F) Organizational priority, leadership support (B, F)
Bridging factors and referrals		
Characteristics of the referral innovation	NA	Presence of SSRLs and customized resource directories (B, F) SSRLs have limited capacity or access to universal databases to maintain resource information (B) Service fees for bidirectional referral and communication (B) Alignment of SSRL with health care and SSO workflows (B, F) Integration of SSRLs in the EHR (F)
Relationships	NA	Bidirectional communication and referral pathways (F) Effective communication and shared goals (F) Quality/trusting relationship (F) Lack of service agreements (B)
Social service context		
Inner setting	NA	Specialized services to support unmet social needs (F) Strong ties in the community (trusting relationships with the people they serve) (F) Long waitlists to access resources and strict eligibility requirements (B) Limited resources (staff, services) to address patients’ needs and keep resource offerings up to date (B) Limited capacity to engage with and maintain SSRLs (B)

Abbreviations: CHWs, community health workers; EHR, electronic health record; NA, not applicable; SDOH, social determinants of health; SSO, social service organization; SSRL, social service referral locator.

a Determinants may be a barrier (B) or facilitator (F), depending on their presence or absence and relative positive or negative effect.

## REVIEW OF SCREENING TOOLS

Kaiser Permanente Washington Health Research Institute collaborated with the Social Interventions Research and Evaluation Network (SIREN) on a systematic review of multidomain screening tools. The review provides an overview of each tool, describes the genesis of the tool, and assesses available evidence on the tool’s psychometric and pragmatic properties. The authors recommend consulting this systematic review to select social risk screening tools most appropriate to one’s setting and population: https://sdh-tools-review.kpwashingtonresearch.org/.

## GUIDE FOR SELECTING A REFERRAL PLATFORM

The authors recommend using the report “Community Resource Referral Platforms: A Guide for Health Care Organizations” to explore the landscape of referral platforms and the experiences of early adopters (16). The guide provides a primer on the key features and functionalities of these technologies, distills lessons learned, and offers recommendations on how to implement a referral platform (for more information, see https://sirenetwork.ucsf.edu/sites/default/files/wysiwyg/Community-Resource-Referral-Platforms-Guide.pdf).

## GUIDES TO IMPLEMENTING SCREENING AND REFERRAL PRACTICES

Participatory and QI approaches may be aided by the following publicly available guides.

- The “Guide to Implementing Social Risk Screening and Referral-Making” (45) provides a five-step roadmap for implementing/improving social risk screening and related activities in primary/ambulatory care settings: https://sirenetwork.ucsf.edu/guide-implementing-social-risk-screening-and-referral-making.
- The “Social Needs Referrals in Primary Care: An Implementation Toolkit” is an interactive decision-making tool kit organized by important social risk referral considerations: preparation (e.g., engaging community-based organizations and assessing clinical and community capacity to address unmet social needs), workflow decisions, staff orientation, program evaluation, and program sustainment: https://sirenetwork.ucsf.edu/tools-resources/resources/social-needs-referrals-primary-care-implementation-toolkit.

## FUTURE ISSUES

1. Further evidence that demonstrates the impact of social needs care on health care quality, cost effectiveness, and patient and population health and equity outcomes is needed to incentivize supportive policies and funding.
2. Sufficient investment in the workforce and infrastructure within clinical and social service organizations and systems is required to further the implementation, sustainability, and impact of social needs care in health care.
3. Improved relationships and interoperable systems across the health care, social service, and public health sectors are needed to ensure that patients’ needs are met and proper evidence is generated.
4. Evidence to inform best practices for screening implementation (e.g., verbally asked by someone they trust versus online surveys; where and when screening is done in the care visit) and ways in which processes should be tailored to reduce biases in reporting is needed, particularly among marginalized populations who face stigma and discrimination.
5. Researchers need more multilevel measures of social risks (50) and strategically leveraged screening data to address SDOH that generate individual risks and structural barriers to accessing assistance (52, 53).
6. Participatory approaches that engage diverse partners within and outside the health care setting to plan for development, implementation, and sustainability should be leveraged prior to launching interventions.
7. While numerous determinants are presented in this review, gaps remain in our understanding of the complex factors that impact the implementation of awareness and assistance interventions in clinical settings (89).
8. Further research is required to generate evidence for effective implementation strategies, their mechanisms (e.g., how they generated the intended impact), and best approaches for tailoring to the context and population.
9. Implementation science must continue efforts to integrate equity into methods and measures (9, 12, 81, 94, 95) and apply these to ensure equitable implementation and outcomes of social needs care.

## Abbreviations

Social determinants of health (SDOH): the conditions in which people are born grow, live, work and age; shaped by the distribution of money, power, and resources
Health equity: “The assurance of the conditions for optimal health for all people. Health disparities will be eliminated when health equity is achieved”
Social risk: specific adverse social conditions that are associated with poor health, e.g., social isolation or housing instability
Social needs: individuals’ perceptions of their most pressing adverse SDOH with which they want assistance
Health disparities: potentially avoidable health differences linked with economic, social, or environmental disadvantage
Health inequities: unfair, unjust, unnecessary, and avoidable differences in health arising from the systemic disadvantage of marginalized groups
Value-based care: financial model that rewards health care providers for the quality of care they provide rather than quantity of care
Social service organizations (SSOs): organizations, including social service agencies and community-based organizations, that provide social services (e.g., utilities and rent assistance) to the public
Awareness: awareness activities identify the social risks/needs and assets of defined patients and populations
Adjustment: activities that focus on altering clinical care to accommodate identified social risks
Assistance: activities that reduce social risk by helping connect patients with relevant social care resources
Advocacy: activities to promote policies that facilitate the creation and redeployment of social resources to address health and social needs
Alignment: activities by a health care system to understand, organize, invest in, and deploy existing community social resources to improve health
Determinants: factors that enable (i.e., facilitators) or inhibit (i.e., barriers) implementation success and impact implementation outcomes
Social service resource locators (SSRLs): digital platform that serves the primary functions of resource directory and referral management. Also referred to as community resource referral platforms
